# Addition of Prebiotic Rice Bran to Ready-to-Use Therapeutic Food Modulated Changes in Body Composition Only of 6–23-Month-Old Children During Treatment for Uncomplicated Acute Malnutrition: The Solutions to Enhance Health with Alternative Treatment (SEHAT) Study

**DOI:** 10.3390/nu18121836

**Published:** 2026-06-06

**Authors:** Silvia Barbazza, Marinka van der Hoeven, Maiza Campos Ponce, Annika M. Weber, Moretta D. Fauzi, Damayanti D. Soekarjo, Elizabeth P. Ryan, Sonia Fortin, Frank T. Wieringa

**Affiliations:** 1 Department of Health Sciences, Faculty of Science, Amsterdam Public Health, Vrije Universiteit Amsterdam, 1081 HV Amsterdam, The Netherlands; m.vander.hoeven@vu.nl (M.v.d.H.); m.camposponce@vu.nl (M.C.P.); m.d.fauzi@vu.nl (M.D.F.); franck.wieringa@ird.fr (F.T.W.); 2 Department of Food Science and Human Nutrition, Colorado State University, Fort Collins, CO 80523, USA; annikamweber@gmail.com (A.M.W.); e.p.ryan@colostate.edu (E.P.R.); 3 Qualisud, University of Montpellier, Avignon University, CIRAD, Institut Agro, IRD, Université de la Réunion, 34394 Montpellier, France; sonia.fortin@ird.fr; 4 Mother and Child Health Department, Mohammad Hoesin Hospital, Palembang 30126, South Sumatera, Indonesia; 5 Child Health Department, Faculty of Medicine, Sriwijaya University, Palembang 30126, South Sumatera, Indonesia; 6 Savica Consultancy, Surabaya 60216, East Java, Indonesia; 7 French National Research Institute for Sustainable Development (IRD), 34394 Montpellier, France

**Keywords:** ready-to-use therapeutic foods, acute malnutrition, body composition

## Abstract

**Background:** Ready-to-use therapeutic foods (RUTFs) have been developed to treat severe acute malnutrition (SAM) in children by promoting rapid weight gain, but the long-term effects have been overlooked. Incorporating prebiotic rice bran into RUTF can enhance balanced weight gain. We hypothesized that children receiving RUTF + rice bran would exhibit increased fat-free mass (FFM) and reduced body fat percentage and abdominal adiposity. **Methods:** A double-blinded randomized controlled trial (ClinicalTrials.gov:NCT05319717) involving 200 children with different degrees of acute malnutrition compared the effectiveness of RUTF with or without rice bran. Children received treatment for 8 weeks, with another 8 weeks of follow-up. Anthropometry, including skinfolds, was collected every 4 weeks. **Results:** Compliance was similar in both groups (~21%). Children aged 6 to 23 months receiving RUTF + rice bran gained more FFM than those receiving RUTF alone (*p* = 0.05 at week 8). Over the 8-week treatment, the fat mass index increased in children receiving RUTF (*p* = 0.02), but not in those receiving RUTF + rice bran (*p* = 0.48), although the increase in body fat percentage was similar (*p* = 0.23). The ratio of abdominal to peripheral skinfolds decreased in both groups during treatment but increased during follow-up, though the difference was not statistically significant. In children aged 24 to 59 months, no significant differences in body composition were observed. The fat-free mass index increased in both groups during treatment but declined afterwards, with significant changes noted in the RUTF + rice bran group. **Conclusions:** The addition of rice bran to RUTF affected body composition changes during treatment only in younger children, where more lean mass was gained and fat mass gain was limited. Differences in intestinal microbiome maturity might underlie this age difference.

## 1. Introduction

Children with acute malnutrition have a notably higher risk of death and therefore require timely access to treatment, including nutritional supplements, such as ready-to-use therapeutic or supplementary foods (RUTFs/RUSFs). Altered body composition in malnourished children contributes to increased mortality risk, partly due to impaired immune function [1]. A study on Cambodian malnourished children showed that children with acute malnutrition have lower fat and fat-free mass (FFM) compared to healthy children, with disproportionately lower fat-free mass [2]. Indeed, it appears that fat mass is relatively spared in the trajectory towards malnutrition [3]. Low levels of fat mass (FM) are associated with low levels of the hormone leptin in malnourished children, a strong predictor of mortality [1]. But lower FFM, and specifically muscle mass, also increases mortality risk, especially during infections [4].

Besides posing an immediate health risk, early-life malnutrition is linked to the onset of nutrition-related NCDs in later life [5]. Specifically, children who have recovered from severe acute malnutrition (SAM) exhibit traits of what is known as thrifty growth, which is associated with future cardiovascular and metabolic disease [6]. Thrifty growth refers to a metabolic adaptation to early-life nutritional deprivation, whereby the body prioritizes essential organ development to maximize survival [7]. Thrifty traits include lower fat-free mass [8], which may indicate low metabolic capacities for optimal functioning for the organism and a reduced capacity to endure the metabolic demands linked to elevated fat mass in adult life [6]. According to Wells (2019) [6], along with FFM, other body composition indicators should be considered markers of metabolic capacity, including total fat mass and peripheral and central abdominal adiposity.

In Indonesia, wasting affects 8.5% of children aged 6–59 months [9]. Import restrictions on RUTFs hamper national treatment efforts. As a result, Indonesia is developing locally produced therapeutic foods that meet national standards. This aligns with broader health goals, as 76% of deaths in the country are due to NCDs [10], highlighting the potential dual benefits of optimized RUTF formulations.

The standard formulation of RUTF for treatment of SAM was developed to prioritize rapid weight gain, but it did not consider the long-term effects on body composition or relapse risk [11]. Recent studies have shown that the etiology of childhood malnutrition is not only a lack of energy, protein, or essential nutrients but also the maturity of the gut microbiome [12,13]. To address these shortcomings, alternative formulations of RUTFs have included whole-food pre- and probiotics aimed at improving gut health, and some studies have shown enhanced nutrient absorption and promising effects on anthropometric outcomes during treatment, with long-lasting effects post-treatment [14,15]. Rice bran represents a particularly promising candidate for such reformulation efforts, given its nutrient profile and local availability in many low- and middle-income countries.

Rice bran, a nutrient-rich by-product of brown rice, contains hundreds of bioactive compounds, including prebiotic fibers, essential fatty acids, amino acids, micronutrients, and phytochemicals [16].

To date, no study has evaluated whether the inclusion of rice bran in RUTF influences body composition recovery during rehabilitation from acute malnutrition, and specifically whether it affects fat-free mass accretion compared to a standard RUTF formulation. While not previously used for acute malnutrition, rice bran has shown promise in preventing enteric infections [17] and supporting growth and microbiota diversity in at-risk populations [18]. Rice bran has demonstrated relevant prebiotic properties. Its dietary fibers have been shown to stimulate the fermentative activity of the human gut microbiota in vitro, increasing the abundance of Bifidobacterium and Lachnospiraceae [19]. Adding rice to local diets in rural Guatemala [20] has also been found to be acceptable and to improve the ability of children and mothers at risk of malnutrition to meet their daily nutrient needs [18]. By enhancing gut integrity and microbial metabolism, rice bran may promote nutrient absorption, support lean mass recovery during nutritional rehabilitation, and mitigate thrifty growth patterns.

This study evaluated the addition of 5% rice bran to a locally produced RUTF in a double-blinded, randomized controlled trial in children with SAM or approaching SAM.

The 5% heat-stabilized rice bran in RUTF + rice bran replaced part of the maltodextrin, skim milk powder, oil, sugar, and wheat flour. A targeted nutrient composition analysis of the RUTF + rice bran [21] revealed significant metabolite fold changes across multiple compound classes, including lipids, amino acids, carbohydrates, vitamins, and xenobiotics, in formulations containing 5%, 7.5%, and 10% rice bran compared with the 0% control. A recent study has demonstrated that the inclusion of rice bran in bakery products can influence technological and sensory properties in a dose-dependent manner [22]. It increased dietary fiber and antioxidant activity, but levels above 15% reduced dough elasticity and negatively affected odor, flavor, and softness. The proportion of rice bran was selected based on nutritional, physical, and sensory criteria informed by a nutrient composition analysis of the product and prior literature. Nutritionally, the target was to maximize fiber and micronutrient contribution while preserving technological and sensory properties.

The primary outcomes of this study [23] were changes in weight, MUAC, and anthropometric *z*-scores over time. At week 4, the PP analysis showed that children receiving RUTF + rice bran had significantly higher weight gain velocity and MUAC velocity compared to RUTF alone, both across all ages and specifically in the 24–59 months age group. No other significant differences were observed between the treatment groups for the other anthropometric measures at the evaluated time points.

As pre-defined in the study protocol, this is an exploratory, hypothesis-generating, secondary outcome analysis aimed to assess changes in body composition over 8 weeks of treatment and 8 weeks post-treatment follow-up in children receiving a local RUTF with or without rice bran. RUTF formulation used in this study had been shown to be acceptable and effective [24]. Drawing on the rationale outlined above, we hypothesized that children receiving RUTF + rice bran would exhibit more favorable body composition recovery than those receiving standard RUTF, including greater fat-free mass accretion, lower body fat percentage, and lower abdominal relative to peripheral fat.

## 2. Materials and Methods

### 2.1. Study Design and Participants

A total of 200 children aged 6–59 months with uncomplicated SAM (WHZ < −3 and/or MUAC < 115 mm, or bilateral edema +/++) or approaching SAM (WHZ < −2.5), after being screened for exclusion criteria [25], were randomly allocated to one of the two interventions. The inclusion of children approaching SAM was necessary to achieve the target sample size within the study timeframe and reflects the continuum of acute malnutrition severity observed in program settings. Stratified baseline data (Table 1) confirm a comparable distribution of SAM children across treatment arms and age groups, suggesting that severity at enrolment is unlikely to have differentially affected body composition outcomes between groups. However, the inclusion of children approaching SAM may have partially attenuated the magnitude of baseline body composition deficits observed, given that SAM is associated with substantially greater FFM and FM deficits than in cases of less severe wasting [3]. Participants were enrolled in Jember District, East Java, Indonesia, between 1 December 2022 and 11 April 2023. The present study is an exploratory, hypothesis-generating, secondary outcome analysis of the SEHAT project, further exploring anthropometric changes during SAM treatment, using body composition data of the children who participated in the trial.

The details of the study’s design and rationale have been previously described [25]. In short, the study aimed to determine the effectiveness of an RUTF with rice bran (RUTF + rice bran) compared with the same RUTF without rice bran (RUTF) in a community setting treatment of uncomplicated SAM, recruiting children with SAM or approaching SAM. For this exploratory secondary outcome analysis, we hypothesized that the children consuming RUTF + rice bran would have changes in body composition that differed from children receiving only RUTF, including increased fat-free mass, lower body fat percentage, and lower abdominal adiposity compared to peripheral adiposity.

Children were randomly assigned to either the intervention group treated with RUTF + rice bran or the control group treated with RUTF. The randomization was done by a researcher (FTW) not involved in the fieldwork, using a computer-generated list with a block size of 8 and two different codes per treatment group. Block size and randomization sequence were not disclosed to the field staff involved in recruitment or follow-up to ensure blinding. Per protocol, the allocation was stratified on age group, 6–23 months and 24–59 months.

### 2.2. Products

The products used in the study were packaged similarly and labeled with a code and flavor. The intervention was kept blinded to researchers, caregivers, and field teams until the completion of the study, including the analysis of primary and secondary outcomes.

The RUTF and RUTF + rice bran were locally produced by the SEAFAST Centre and the Department of Community Nutrition at Bogor Agricultural University, Indonesia. Both products underwent testing to meet WHO standards on maximum toxin levels and complied with the Codex Alimentarius hygienic practice code (CAC/RCP 21-197). Full product details have been reported previously [25], and they are reported in Supplementary Table S1.

### 2.3. Intervention

Dietary treatment for acute malnutrition followed Indonesia’s national guidelines. The RUTF intervention lasted for eight weeks, followed by eight weeks of post-treatment follow-up. After enrolment, trained fieldworkers collected anthropometric measurements, including weight, height, MUAC, edema, and skinfold thickness at four sites (biceps, triceps, subscapular, suprailiac). At baseline, caregivers completed a questionnaire on birth date, birth weight, birth mode, and infant sex, with age calculated from the recorded date of birth at each visit. They also provided a 1-week supply of RUTF based on the child’s body weight, in accordance with WHO standards, and provided instructions for administering the RUTF.

Weight was measured to the nearest 0.1 kg using SECA scales (Seca 874 Flat and 874DR), taken twice and repeated a third time if the first two differed by >0.2 kg. Length/height was measured to the nearest 0.1 cm using a ShorrBoard: children <24 months or <87 cm were measured in a supine position, and older/taller children were measured whilst standing. All length/height measurements were taken twice and repeated if they differed by >0.2 cm. MUAC was measured on the left arm with a color-coded standard tape to the nearest 0.1 mm, taken twice and repeated if the first two differed by >0.2 cm. Edema was assessed by checking for a shallow imprint after pressing both feet for 3 s. Skinfolds (triceps, biceps, subscapular, suprailiac) were measured on the left side using Lange calipers, taken twice, and repeated if measurements differed by >2 mm.

Monitoring visits occurred weekly during the eight-week RUTF intervention. Edema, MUAC, and weight were measured weekly, while skinfolds and height were reassessed at weeks 4 and 8, and again at follow-up visits (weeks 12 and 16). Fieldworkers recorded caregiver reports of daily RUTF intake, and any remaining RUTF was weighed to document weekly consumption. Any side effect defined according to the protocol [25] was also monitored and recorded.

### 2.4. Outcomes

The body composition outcomes considered in this analysis include body fat percentage (BFP), fat mass, and fat-free mass, expressed in kilograms.

The BFP was calculated for females and males using specific body composition prediction equations based on all four skinfolds and adjusted for children’s height (Supplementary Table S2). These equations were tested in children aged 3 to 21 months old with syndromes affecting growth and body composition, such as Down syndrome, Crohn’s disease, and steroid-sensitive nephrotic syndrome, and showed excellent agreement with DXA measurements [26]. Fat mass was calculated as total weight × body fat percentage/100, and fat-free mass as total weight minus fat mass. The fat-free mass index (FFMI) and fat mass index (FMI) were obtained by dividing FFM and FM (kg) by height^2^ (kg/m^2^). Subcutaneous fat levels were measured via skinfolds (mm) and converted to age- and sex-specific *z*-scores using the zscorer package (v0.3.1) in R. Abdominal skinfolds were defined as the sum of suprailiac and subscapular measures, and peripheral skinfolds as the sum of triceps and biceps measurements.

Weight-for-height (WHZ), weight-for-age (WAZ), and height-for-age (HAZ) *z*-scores were computed using the WHO 2006 growth standards in R, with zscorer package (version 0.3.1).

The Infant and Child Feeding Index (ICFI), a unique index summarizing feeding practices for children 6 to 59 months of age, was calculated according to an adaptation of the method described by Arimond and Ruel [27]. Building on the original method, children aged 36–59 months were also included (Supplementary Table S3). Based on their dietary score, children were classified as having a poor diet (0–2), borderline diet (3–4), or acceptable diet (5–6).

### 2.5. Sample Size

Although randomization was stratified by age group (6–23 and 24–59 months), as pre-specified in the study protocol, the power calculation was performed for the overall sample. The sample size of 100 children per intervention was calculated for the main study to detect a 20% difference in weight gain between the control and rice bran products, with a significance level of 0.05 and a power of 0.80 [25]. Although the sample size was not initially calculated to detect differences in body composition outcomes, previous studies using skinfold thickness to assess vitamin D supplementation’s effect on newborns and zinc supplementation’s effect on body composition in stunted children showed significant differences in skinfold measurements with equal or lower sample sizes [28,29].

### 2.6. Statistical Analysis

Data were cleaned and analyzed in R (version 4.2.2, macOS). Baseline numerical variables were tested for normality using the Shapiro–Wilk test and, as all were non-normal (*p* < 0.05), the Wilcoxon rank-sum test was used; categorical variables were compared using Pearson’s Chi-square test. Continuous data are reported as median [IQR], and categorical data as percentages (n).

Measures of skinfold thickness, abdominal-to-peripheral skinfold ratio, BFP, FM, and FFM were analyzed using linear mixed models to account for repeated measures. No correction for multiple comparisons was applied, given the exploratory nature of this analysis.

Fixed effects included treatment (RUTF + rice bran, RUTF), time (weeks 0, 4, 8, 12, 16), sex, age, and treatment-by-time interactions, with child ID as a random effect. Models were run for all participants as well as stratified by age group (6–23 and 24–59 months). Statistical significance was set at 0.05. Tukey’s post hoc tests compared body composition outcomes across treatments within each week and across weeks within each treatment. Endpoints were analyzed using the complete case analysis approach for all enrolled children, with no imputation of missing body composition data. The results are presented as means and standard deviation (SD). A sensitivity analysis, using last observation carried forward (LOCF), was also conducted to assess the robustness of the findings to missing data.

The same model was used to assess the difference from baseline at each time point (weeks 4, 8, 12, and 16) in skinfold thickness, the ratio of abdominal and peripheral skinfolds, BFP, FM, FFM, FMI, and FFMI.

The SEHAT study protocol had Colorado State University IRB approval (IRB #1823, OHRP FWA00000647) and approval through the Medical and Health Research Ethical Committee (MHREC) at the Faculty of Medicine, Public Health and Nursing, Universitas Gadjah Mada in Yogyakarta, Indonesia Ref. No.: KE/FK/0546/EC/2022 and KE/FK/0703/EC/2023. The trial is registered on clinicaltrials.gov NCT05319717. Informed consent was obtained from participants’ caregivers prior to children’s enrolment into the study.

## 3. Results

**Figure 1:**
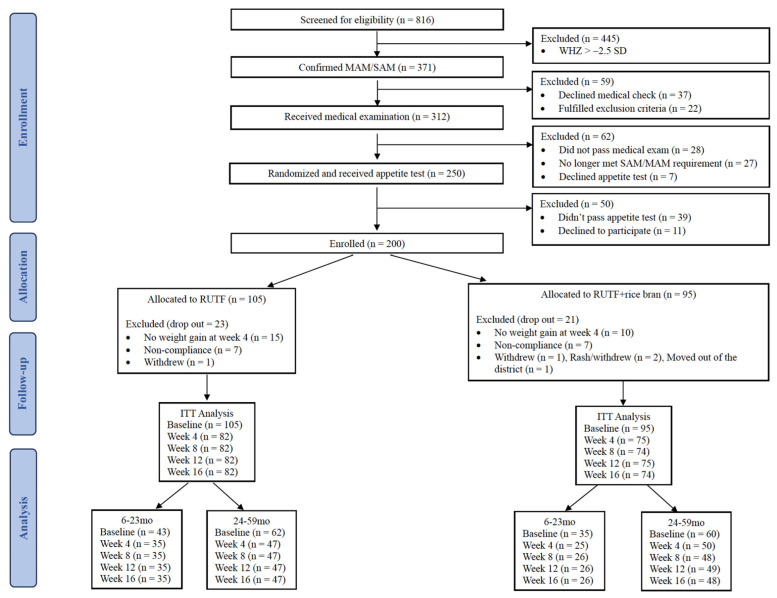
SEHAT CONSORT flow.

Of 816 children screened, 250 children were randomized, and 200 were eligible, of which 95 were allocated to RUTF + rice bran and 105 to RUTF. Group sizes differed because randomization occurred before the appetite test, leading to exclusions for those who failed it. Importantly, exclusion following failed appetite test occurred across both arms and was unrelated to the study intervention, as children had not yet received any treatment at the time of the appetite test. This post-randomization exclusion is therefore unlikely to have introduced systematic bias in either direction. By week 16, 156 children completed the study (74 RUTF + rice bran; 82 RUTF). Twenty-five children dropped out by week 4 per protocol due to failure to gain weight, or did not consume RUTF for 2 weeks, and were referred to local clinics per protocol [25]. A further 18 withdrew because of non-compliance, rash, or voluntary withdrawal, and one child missed measurements at weeks 8 and 16 (Figure 1). Dropout rates did not differ between groups. Compliance with the prescribed RUTF supplementation was low but comparable between treatment arms (~21% in both groups).

Table 1 presents the demographic and anthropometric data by age group and treatment. Although the median triceps skinfold *z*-score was statistically significantly higher in the RUTF group at baseline, no significant differences between treatment arms were observed in FFM, FM, or skinfold ratio at enrolment (Table 1), indicating that this difference was not accompanied by a meaningful imbalance in overall body composition.

The average compliance rate was low at 20.9% for the RUTF group and 21.2% for the RUTF + rice bran group during the 8-week study (Supplementary Table S4). All subsequent findings should be interpreted in this context. Compliance was defined as the proportion of the prescribed RUTF dose consumed, calculated by comparing the amount of RUTF distributed—based on child weight at baseline and adjusted at each weekly visit (Supplementary Table S5)—with the amount remaining as determined by caregiver diaries and weighing of unconsumed sachets [25]. No adverse events were reported in either group. No significant differences were observed in the Infant and Child Feeding Index analysis or breastfeeding practices at weeks 8 and 16 between the treatment groups (Supplementary Table S6).

### 3.1. Children Aged 6–59 Months

Table 2 presents body composition results for all ages at baseline, treatment, and follow-up. BFP increased slightly during treatment and then stabilized. In the RUTF + rice bran group, FM rose by 240 g by end of treatment and remained stable, whereas FM in the RUTF group continued increasing post-treatment, reaching +280 g by week 16.

FFM increased more in the RUTF group by week 16, with a 100 g difference versus RUTF + rice bran, although this difference was not statistically significant. FMI rose similarly in both groups, reaching 0.09 kg/m^2^ in RUTF + rice bran and 0.12 Kg/m^2^ in RUTF by follow-up at week 16. FFMI increased slightly more (0.01 Kg/m^2^) in the RUTF + rice bran group during treatment, but without statistical significance (*p* = 0.33). During follow-up, FFMI continued rising in the RUTF group but declined toward baseline value in the RUTF + rice bran group. Significant FFMI changes were observed only in the RUTF + rice bran group in the all-ages sample and among children aged 24–59 months (Tukey comparisons, Supplementary Table S7).

The single measurements of the abdominal-to-peripheral skinfold ratio did not differ between groups at any time point. However, the ratio changes from baseline diverged: the RUTF group showed decreases at weeks 8 and 16, while the RUTF + rice bran group increased, producing a significant difference at week 16 (*p* = 0.02) (Supplementary Figure S1). Individual skinfolds indicated slightly greater abdominal fat gain in the RUTF group, including increases in suprailiac skinfolds after treatment. The ratio difference reflects a larger rise in biceps skinfolds from week 0 to week 16 in the RUTF group compared with the RUTF + rice bran group (*p* = 0.03) (Supplementary Figure S1). Although the difference in biceps skinfold thickness at week 16 reached statistical significance (4.64 vs. 4.88 mm, *p* = 0.02), the absolute difference of ~0.24 mm falls below the repeatability threshold of Lange calipers (~2 mm) and should therefore be interpreted with caution, as it is unlikely to reflect a physiologically meaningful difference in peripheral fat deposition.

### 3.2. Children Aged 6–23 Months

Table 3 shows body composition results for children aged 6 to 23 months at baseline, end of treatment, and follow-up. Children treated with RUTF showed a greater BFP at the end of treatment and follow-up (0.8%) compared to those treated with RUTF + rice bran (0.3%), although this difference was not significant. No differences were found in FM accumulation between the treatment groups. Over the 8-week treatment, children receiving RUTF + rice bran gained more fat-free mass (370 g) than those on RUTF alone (210 g) (*p* = 0.05), a difference persisting up to the end of follow-up. This borderline finding should be interpreted cautiously and requires confirmation in future adequately powered studies.

There were no significant differences in FMI and FFMI between treatment groups at any time point. Nevertheless, FMI seemed to rise more in the RUTF group during treatment. During follow-up, it further increased in the RUTF + rice bran group while slightly decreasing in the RUTF group. During treatment, FFMI increased in the RUTF + rice bran group, remaining steady in the RUTF group, but this increase did not persist afterwards.

The ratio of abdominal to peripheral skinfolds decreased in both groups during treatment but increased for both during follow-up, although the difference was not statistically significant. In the RUTF + rice bran group, this may relate to the decrease in biceps skinfolds and the increase in subscapular skinfolds during the follow-up period. In children 6–23 months, the triceps skinfold difference from baseline to week 8 was greater in the RUTF group than in the RUTF + rice bran group (0.74, ±SE 0.37 *p* = 0.03) (Supplementary Figure S1).

### 3.3. Children Aged 24 to 59 Months

Table 4 presents body composition results for children aged 24 to 59 months, recorded at baseline, end of treatment, and follow-up completion period. No statistically significant differences were found in the main body composition outcomes analysis.

Compared to the RUTF + rice bran group, the RUTF group showed a 5 g increase in FM during treatment, and this higher level continued throughout the follow-up period but never reached statistical significance. Children on RUTF gained more fat-free mass during treatment and follow-up. FMI increased in the RUTF + rice bran group during treatment but decreased in both groups during follow-up.

FFMI rose in both groups during treatment but declined afterwards. Tukey’s comparison in the week-by-treatment analysis (Supplementary Table S7) showed significant FFMI changes only in the RUTF + rice bran group during the treatment period.

The ratio of abdominal to peripheral skinfolds increased in both groups during treatment but continued to rise only in the RUTF + rice bran group post-treatment. This difference, although not statistically significant, may be due to a 0.10 mm decrease in the triceps skinfold in this group, while the RUTF group experienced persistent increases. In children aged 24–59 months, biceps skinfolds increased more from week 0 to week 16 in the RUTF group than in the RUTF + rice bran group (*p* = 0.04) (Supplementary Figure S1).

## 4. Discussion

Data on body composition in children recovering from SAM are scarce, and our results show that modest changes in the composition of RUTF can have significant effects on body composition changes during treatment, with differences persisting until at least 8 weeks post-treatment. Our findings also highlight distinct patterns based on age, with effects of the prebiotic rice bran on body composition being considerably different between younger and older children. These patterns are relevant not only for understanding the nutritional impacts of these interventions but also for informing dietary recommendations for children in malnutrition recovery contexts.

### 4.1. Fat Mass and Fat-Free Mass

Across all age groups, FM increased earlier in the RUTF + rice bran and then stabilized, whereas FM in the RUTF-only group continued to rise post-treatment. Similarly, in children aged 24–59 months, rice bran had a more pronounced effect on BFP during treatment, which diminished by the follow-up phase. This aligns with previous studies [2,30], which found an increase in fat mass during treatment, and suggests that rice bran may help regulate long-term fat mass gain. This finding is notable since fat accumulation in infancy has been associated with a higher risk of developing cardiovascular diseases in life [31].

Among children aged 6–23 months, those receiving RUTF + rice bran gained more lean mass, but this difference, which was not statistically significant, did not persist through the follow-up period. This suggests that the addition of rice bran to RUTF may be particularly beneficial in promoting lean tissue growth in younger children. Although micronutrient status was not analyzed in this study, we hypothesize that the prebiotic and micronutrient-rich properties of rice bran may have supported enhanced nutrient absorption [32] and lean mass accretion in younger children, whose gut microbiota and absorptive capacity are still developing; this remains speculative and requires confirmation in future studies measuring micronutrient status and gut microbiota outcomes directly. Previous studies have shown that deficiencies in selenium, carotenoids, and vitamin E are associated with reduced muscle mass and strength in elderly populations [25] and animal models [33]. Micronutrient supplementation, including iron, zinc, and vitamin A, has been shown to improve fat-free mass and linear growth in children [34]. A recent review on micronutrient status in children with SAM reported a high prevalence of vitamin A deficiency (25–78%) and iron deficiency anemia (40–94%), alongside lower serum selenium. Zinc deficiency is also common in LMICs (30–70%) [35]. The first two years of life offer a vital opportunity to incorporate pre- or probiotics into the treatment of acute malnutrition. This period represents a critical window of gut microbiome maturation, during which microbial diversity and functional capacity develop rapidly, the microbiota remains highly responsive to dietary exposures such as prebiotics, and key metabolic and immune pathways are being established [36].

### 4.2. Fat Mass Index and Fat-Free Mass Index

In children of all ages, FMI increased in both groups during treatment, which subsequently decreased during follow-up, mirroring the trend observed for BFP accumulation. When examining the data by age group, it was found that children aged 6–23 months who were treated with RUTF + rice bran continued to increase FMI even after treatment. In the same age category, FFMI rose during the treatment phase in the RUTF + rice bran group, while it remained stable in the RUTF group, with this effect not persisting post-treatment. The results align with a study of Cambodian children aged 6 to 15 months, suggesting that undernourished children retain body fat at the expense of fat-free mass tissue [37]. These observations also indicate that for younger children, RUTF + rice bran may offer a more balanced approach to promote both fat and lean mass accumulation, although its impact diminishes over time for lean mass gain.

Over the 8-week treatment period, FFMI increased in both groups among children aged 24–59 months; however, statistically significant gains from baseline were observed only in the RUTF + rice bran group at week 4 (Supplementary Table S7), with no significant within-group changes detected in the RUTF-alone group at any time point, suggesting that lean mass accrual during treatment was specific to rice bran supplementation. These gains did not persist following treatment cessation, with FFMI returning to baseline values in both groups by the end of the follow-up.

These findings are particularly relevant when considering that participants in the SEHAT study exhibited a mean FM and FMI across all age groups at baseline and during the study period that were higher than those of a similar cohort in Cambodia [2] and India [38], and compared to healthy children in Japan [39], while FFM and FFMI were lower (Table 2, Table 3 and Table 4). A study on Indonesian growth standards shows that the growth patterns of Indonesian children resemble those of healthy Japanese children [40]. A low FFM indicates reduced metabolic capacity, impairing the ability to manage metabolic demands from increased adiposity [6,41]. Research shows that people with better metabolic capacity have a lower chance of developing non-communicable diseases, while those with a heavier metabolic load are at a higher risk [41].

### 4.3. Abdominal-to-Peripheral Skinfold Ratios and Fat Distribution

Differences between the groups were observed in the abdominal to peripheral skinfold ratios of children of all ages, with the RUTF + rice bran group exhibiting a statistically significantly higher increase at each time point during follow-up. This difference is no longer significant when the data are disaggregated by age group, which may be explained by the reduced sample size. A higher ratio suggests a shift toward more centralized fat accumulation in the RUTF + rice bran group. However, consistent with previous studies [3], this difference appears to be associated with a higher increase in peripheral mass in children treated with RUTF compared to an increase in abdominal fat mass accumulation in the RUTF + rice bran group. In both groups, at baseline, during, and after treatment, the ratio remains below one, indicating that a higher proportion of fat mass accumulated in the peripheral areas compared to the abdominal region throughout the study and for both treatment groups. Researchers [42,43] suggest that abdominal fat can increase the risk of developing NCDs, while peripheral fat can protect against it.

### 4.4. Strengths and Limitations

A double-blind, randomized controlled trial is the gold standard for assessing the effectiveness of rice bran-based therapeutic food (RUTF) and reducing potential bias. Moreover, children were closely followed during treatment and follow-up, allowing for the collection of high-quality data.

The study provides new insights into rice bran as a potential novel ingredient for optimizing the treatment of severe acute malnutrition, with potential beneficial effects on body composition.

This study has several limitations. First, the power calculation was conducted for the primary outcomes and the overall cohort analysis, without considering sub-analyses of children divided by age groups. The results of a post hoc power analysis indicated that, with approximately 100 children per treatment arm, the study had 80% power to detect a standardized effect size of Cohen’s d = 0.40 (small-to-medium effect) for body composition outcomes at a significance level of 0.05. However, the age-stratified subgroup analyses included only 35–43 children per arm in the 6–23 months group, resulting in these subgroup analyses being underpowered. The detectable effect sizes reflect the study’s statistical capacity, not the magnitude of the observed effects. Because the study was not powered specifically for body composition outcomes, the findings should be interpreted as exploratory. This may have limited the ability to detect statistically significant differences within subgroups and should be considered when interpreting the age-stratified findings. Future confirmatory trials should be powered specifically for body composition outcomes in age-stratified subgroups, with sample sizes adjusted for the compliance levels observed in community-based settings (~21%), which would require substantially larger recruitment targets than those used in the present study. The dropout rates were high (22% by week 4), largely due to protocol-mandated discharge following insufficient weight gain. To assess whether dropout introduced differential bias between treatment arms, we compared baseline characteristics of children lost to follow-up against those who completed the study. Children lost to follow-up did not differ significantly from completers in any outcome or characteristics (Supplementary Table S8), and dropout rates were comparable across treatment arms. Furthermore, to assess the robustness of the findings to missing data, a sensitivity analysis was conducted using last observation carried forward imputation for all body composition outcomes across all age groups. The results were broadly consistent with the complete case analysis. Notably, the significant treatment by time interaction (Supplementary Table S9) for the fat-free mass index observed in the primary analysis, both across all ages and in the 24–59 months subgroup, remained statistically significant under LOCF, supporting the robustness of this finding. However, two differences that reached statistical significance in the primary analysis, the abdominal-to-peripheral skinfold ratio and biceps skinfold thickness at week 16 in children aged 24–59 months, were no longer significant under LOCF (*p* = 0.11 and *p* = 0.36, respectively) (Supplementary Table S10), suggesting that these findings were sensitive to the handling of missing data and should be interpreted with caution. Also, compliance with RUTF appears low (~21% in both groups) but consistent with other regional studies [44,45]. Significantly, this trial was conducted in a real-world environment where adherence was not as strictly monitored as in clinical settings. To incentivize product consumption, the flavor of the RUTF was changed at week 4 (from chocolate or vanilla or vice versa). While compliance appears low, this figure requires important contextualization. The quantity of RUTF provided was calculated according to WHO recommendations for a theoretical weight gain of 5–10 g/kg/day [23], which substantially exceeds the weight gain rates of 1.5–3 g/kg/day typically observed in community-based programs in Southeast Asia [44,45]. Critically, the fact that all children gained weight during the study, which would be metabolically impossible if RUTFs were the sole energy source at 21% consumption, strongly suggests that children were concurrently consuming their traditional diet, with RUTF acting as a nutritional supplement rather than a sole source of nutrition. This interpretation is supported by energy balance calculations: assuming a concurrent traditional diet providing approximately 120 kcal/kg/day, the observed weight gain of approximately 2 g/kg/day is entirely consistent with the available energy intake. Furthermore, the absolute increases in fat-free mass observed across both groups indicate that type-2 nutrient availability was not a limiting factor. Nevertheless, the reduced effective rice bran exposure introduces uncertainty about whether the dose consumed was sufficient to produce microbiome-mediated effects on body composition, and it is plausible that higher adherence to the rice bran-enriched formulation could have produced more robust differences between treatment arms. Future studies should incorporate objective compliance monitoring and pre-specify dose–response analyses to address this question. An additional limitation of the study was that it relied solely on skinfold measurements for assessing body composition, which can introduce errors as they may not accurately represent the total body fat, considering that a significant portion of body fat is internal [46]. Although the Wendel et al. (2017) [26] skinfold prediction equation was not validated specifically in acutely malnourished children, it was considered the most appropriate available option given its validation in children with conditions sharing relevant biological characteristics with this population, including impaired growth and altered body composition. An additional limitation concerns the inclusion of children “near SAM” (weight-for-height *z*-score below −2.5), which may have diluted the observed effect compared to a sample including only children with strict SAM (weight-for-height *z*-score below −3).

## 5. Conclusions

These exploratory findings should be interpreted with caution given the study limitations described above, but they provide preliminary evidence that justifies further investigation in future adequately powered trials. The present study suggests that younger children may benefit more from the addition of rice bran to RUTF, showing greater lean mass gain without excessive fat accumulation. These age-specific effects underscore the need for tailored nutritional interventions in malnutrition recovery. Future studies integrating body composition assessment with metabolomic profiling, protein balance analyses, and gut microbiota profiling are needed to elucidate the mechanisms underlying the effects of rice bran RUTF on lean mass gain in acutely malnourished children.

As the third largest rice producer, Indonesia provides abundant rice bran that can be utilized in community health initiatives. Rice bran is an economical, nutrient-rich component that is effective in addressing malnutrition. Still, further research is necessary to understand long-term metabolic consequences and explore the underlying mechanisms behind the differential effects of RUTF and RUTF + rice bran. These outcomes support government efforts to scale acute malnutrition outpatient programs with locally produced RUTF, informing current policy for product standardization in acute malnutrition treatment.

## Figures and Tables

**Table 1:** Characteristics of children at enrollment ^1^.

Characteristics	6–23 Months of Age		24–59 Months of Age	
	RUTF + Rice Bran (n = 35)	RUTF (n = 43)	RUTF + Rice Bran (n = 60)	RUTF (n = 62)
**Sociodemographic characteristics**				
Female, n (%)	13 (37%)	17 (40%)	25 (42%)	28 (45%)
Age, months (IQR)	20.3 (15.6, 21.9)	16.6 (12.4, 20.0)	42 (36.0, 51.0)	40 (29.0, 51.0)
Age of biological mother, year (IQR)	30 (28, 36)	28 (23, 31)	30.0 (26, 33)	29.5 (25, 35)
Mother education, years (IQR)	10 (6, 12)	12 (9, 12)	9 (6, 12)	9 (6, 12)
Age of biological father (IQR)	35 (31, 40)	31 (27, 37)	34 (30, 39)	35 (30, 40)
Father education, years (IQR)	9 (6, 12)	12 (9, 12)	9 (6, 12)	9 (6, 12)
Childbirth weight (g) (IQR)	2700 (2440, 2970)	2670 (2200, 3100)	2700 (2480, 2900)	2700 (2250, 3000)
Low birth weight ^2^ n (%)	10 (29%)	14 (33%)	15 (25%)	21 (34%)
Currently breastfeeding, n (%)	20 (57%)	31 (72%)	5 (5.3%)	7 (11%)
**ICFI** n (%)				
Acceptable	31 (89%)	33 (77%)	53 (88%)	55 (89%)
Borderline	4 (11%)	10 (23%)	6 (10%)	6 (9.7%)
Poor	-	-	1 (2%)	1 (1.6%)
**Anthropometric characteristics**				
Subscapular skinfolds, mm (IQR)	4.00 (4.00, 5.00)	4.00 (4.00, 5.00)	5.00 (4.00, 5.00)	5.00 (4.00, 5.00)
Suprailiac skinfolds, mm (IQR)	3.00 (3.00, 4.00)	3.50 (3.00, 4.00)	3.50 (3.00, 4.00)	4.00 (3.00, 4.00)
Biceps skinfolds, Mm (IQR)	4.00 (4.00, 5.00)	4.00 (3.25, 5.00)	5.00 (4.00, 5.00)	4.00 (4.00, 5.00)
Triceps skinfolds, mm (IQR)	5.00 (5.00, 7.00)	5.00 (4.25, 6.00)	6.00 (5.00, 7.00)	6.00 (5.13, 7.00)
Fat mass, kg (IQR)	2.44 (2.23, 2.61)	2.35 (2.08, 2.56)	2.97 (2.71, 3.22)	2.90 (2.65, 3.26)
Fat-free mass, kg (IQR)	4.62 (4.21, 5.11)	4.59 (4.16, 4.91)	6.69 (5.95, 7.54)	6.50 (5.63, 7.49)
Body fat percentage (IQR)	34.2 (33.1, 35.2)	34.2 (32.3, 35.5)	30.6 (29.0, 32.9)	30.9 (29.3, 32.4)
Ratio abdominal skinfolds/peripheral skinfolds (IQR)	0.85 (0.68, 0.90)	0.88 (0.78, 0.98)	0.80 (0.68, 0.89)	0.80 (0.69, 0.90)
Mid-upper arm circumference, cm (IQR)	12.4 (12.0, 12.9)	12.5 (11.9,12.8)	13.0 (12.6, 13.5)	13.0 (12.6, 13.5)
Weight, kg (IQR)	7.20 (6.50, 7.75)	6.90 (6.35, 7.40)	9.55 (8.80, 10.80)	9.45 (8.23, 10.90)
Length or height, cm (IQR)	74.2 (70.3, 77.3)	72.5 (69.2, 75.4)	89 (84.0, 92.0)	87 (82.0, 94.0)
SAM, <−3 weight-for-height *z*-score n (%)	17 (49%)	27 (63%)	25 (48%)	20 (32%)
Stunted, <−3 height-for-age *z*-score n (%)	15 (43%)	18 (42%)	23 (38%)	17 (28%)
Subscapular skinfolds for age *z*-score (IQR)	−2.50 (−2.80, −1.18)	−2.52 (−3.15, −1.17)	−0.99 (−2.14, −0.72)	−1.01 (−2.18, −0.86)
Triceps skinfolds for age *z*-score (IQR)	−2.20 (−2.72, −0.50)	−2.27 (−3.12, −1.29)	−1.25 (−2.18, −0.61)	−1.27 (−1.88, −0.53)
Weight-for-height *z*-score (IQR)	−2.98 (−3.32, −2.75)	−3.10 (−3.30, −2.80)	−2.97 (−3.21, −2.77)	−2.91 (−3.21, −2.77)
Weight-for-age *z*-score (IQR)	−3.43 (−4.05, −3.15)	−3.51 (−3.78, −3.06)	−3.68 (−3.86, −3.27)	−3.50 (−3.90, −3.17)

^1^ Values are expressed as n (%) or medians and interquartile ranges. Wilcoxon rank sum test for continuous variables. Pearson’s Chi-squared test for categorical variables. ^2^ Low birth weight is defined as <2500 g.

**Table 2:** Body composition in children aged 6 to 59 months at baseline, week 8, and week 16 of the study ^1^.

Children Aged 6–59 Months									
	Week 0			Week 8			Week 16		
Outcome	RUTF+ Rice Bran	RUTF	*p*-Value ^2^	RUTF+ Rice Bran	RUTF	*p*-Value ^3^	RUTF+ Rice Bran	RUTF	*p*-Value ^3^
BFP, %	31.00 (±3.08)	31.10 (±3.05)	0.21	31.9 (±2.85)	31.9 (±3.10)	0.76	31.5 (±2.96)	31.7 (±3.37)	0.47
FM, Kg	2.67 (±0.41)	2.60 (±0.45)	0.88	2.91 (±0.46)	2.83 (±0.49)	0.53	2.91 (±0.49)	2.88 (±0.48)	0.50
FFM, Kg	6.05 (±1.39)	5.89 (±1.55)	0.07	6.29 (±1.34)	6.19 (±1.66)	0.13	6.43 (±1.36)	6.37 (±1.66)	0.83
FMI, Kg m^2^	3.90 (±0.48)	3.93 (±0.47)	0.39	4.09 (±0.49)	4.11 (±0.50)	0.86	3.99 (±0.51)	4.05 (±0.55)	0.54
FFMI, Kg m^2^	8.65 (±0.49)	8.69 (±0.46)	0.25	8.73 (±0.51)	8.76 (±0.48)	0.18	8.67 (±0.50)	8.71 (±0.48)	0.56
Ratio abdominal vs. peripheral skinfolds	0.79 (±0.15)	0.82 (±0.16)	0.20	0.81 (±0.14)	0.82 (±0.16)	0.17	0.84 (±0.17)	0.83 (±0.15)	0.04
Subscapular skinfolds, mm	4.61 (±0.85)	4.50 (±0.86)	0.53	4.93 (±1.02)	4.88 (±0.91)	0.92	5.00 (±0.97)	5.00 (±0.97)	0.84
Suprailiac skinfolds, mm	3.64 (±0.98)	3.68 (±0.86)	0.66	4.22 (±0.97)	4.08 (±0.90)	0.20	4.21 (±0.90)	4.16 (±0.89)	0.51
Triceps skinfolds, mm	6.04 (±1.35)	5.83 (±1.36)	0.51	6.62 (±1.69)	6.29 (±1.44)	0.80	6.68 (±1.64)	6.44 (±1.54)	0.91
Biceps skinfolds, mm	4.56 (±1.07)	4.35 (±1.08)	0.22	4.95 (±1.13)	4.92 (±1.08)	0.30	4.64 (±1.08)	4.88 (±1.23)	0.02

^1^ Data are reported as means (±SD). Statistical significance level *p* < 0.05. ^2^ Comparison between means at baseline. ^3^ Tukey pairwise comparisons, treatment, and week interaction.

**Table 3:** Body composition in children aged 6 to 23 months at baseline, week 8, and week 16 of the study ^1^.

Children 6–23 Months Old									
	Week 0			Week 8			Week 16		
Outcome	Rutf+ Rice Bran	RUTF	*p*-Value ^2^	RUTF+ Rice Bran	RUTF	*p*-Value ^3^	RUTF+ Rice Bran	RUTF	*p*-Value ^3^
BFP, %	33.3 (±2.30)	32.9 (±2.50)	0.43	33.3 (±1.87)	33.7 (±2.42)	0.23	33.6 (±2.34)	33.7 (±2.43)	0.55
FM, Kg	2.31 (±0.27)	2.24 (±0.28)	0.79	2.50 (±0.37)	2.42 (±0.31)	0.83	2.60 (±0.36)	2.53 (±0.30)	0.77
FFM, Kg	4.65 (±0.64)	4.57 (±0.61)	0.60	5.02 (±0.73)	4.78 (±0.63)	0.05	5.16 (±0.79)	4.99 (±0.66)	0.59
FMI, Kg/m^2^	4.32 (±0.36)	4.30 (±0.34)	0.63	4.38 (±0.39)	4.46 (±0.36)	0.46	4.40 (±0.36)	4.44 (±0.39)	0.76
FFMI, Kg/m^2^	8.66 (±0.49)	8.76 (±0.47)	0.37	8.77 (±0.54)	8.77 (±0.49)	0.10	8.67 (±0.52)	8.74 (±0.50)	0.33
Ratio abdominal vs. peripheral skinfolds	0.79 (±0.17)	0.86 (±0.14)	0.08	0.77 (±0.15)	0.80 (±0.16)	0.06	0.83 (±0.20)	0.84 (±0.14)	0.22
Subscapular skinfolds, mm	4.50 (±0.85)	4.43 (±0.97)	0.90	4.43 (±0.84)	4.59 (±0.90)	0.40	4.91 (±0.86)	4.96 (±0.86)	0.69
Suprailiac skinfolds, mm	3.52 (±1.03)	3.61 (±0.76)	0.60	3.93 (±0.98)	3.86 (±0.83)	0.52	4.20 (±0.78)	4.04 (±0.74)	0.35
Triceps skinfolds, mm	5.95 (±1.53)	5.24 (±1.28)	0.15	6.26 (±1.66)	6.09 (±1.57)	0.06	6.67 (±1.87)	6.14 (±1.74)	0.41
Biceps skinfolds, mm	4.50 (±1.05)	4.26 (±1.09)	0.49	4.87 (±0.99)	4.77 (±1.09)	0.64	4.74 (±1.12)	4.91 (±1.09)	0.18

^1^ Data are reported as means (±SD). Statistical significance level *p* < 0.05. ^2^ Comparison between means at baseline. ^3^ Tukey pairwise comparisons, treatment, and week interaction.

**Table 4:** Body composition in children aged 24 to 59 months at baseline, week 8, and week 16 of the study ^1^.

Children 24–59 Months Old									
	Week 0			Week 8			Week 16		
Outcome	RUTF + Rice Bran	RUTF	*p*-Value ^2^	RUTF + Rice Bran	RUTF	*p*-Value ^3^	RUTF+ Rice Bran	RUTF	*p*-Value ^3^
BFP, %	29.9 (±2.80)	29.9 (±2.78)	0.41	31.2 (±2.99)	30.5 (±2.85)	0.72	30.5 (±2.70)	30.2 (±3.20)	0.70
FM, Kg	2.84 (±0.36)	2.84 (±0.37)	0.68	3.09 (±0.43)	3.14 (±0.41)	0.62	3.05 (±0.39)	3.15 (±0.37)	0.59
FFM, Kg	6.73 (±1.13)	6.77 (±1.35)	0.11	6.87 (±1.15)	7.24 (±1.37)	0.63	7.00 (±1.16)	7.40 (±1.41)	0.63
FMI, Kg/m^2^	3.69 (±0.38)	3.68 (±0.39)	0.50	3.96 (±0.48)	3.85 (±0.41)	0.56	3.81 (±0.46)	3.77 (±0.49)	0.62
FFMI, Kg/m^2^	8.65 (±0.49)	8.64 (±0.46)	0.61	8.72 (±0.51)	8.74 (±0.47)	0.79	8.67 (±0.49)	8.70 (±0.46)	0.85
Ratio abdominal vs. peripheral skinfolds	0.79 (±0.14)	0.80 (±0.17)	0.99	0.82 (±0.14)	0.83 (±0.15)	0.64	0.84 (±0.16)	0.82 (±0.16)	0.29
Subscapular skinfolds, mm	4.66 (±0.85)	4.55 (±0.78)	0.47	5.16 (±1.02)	5.11 (±0.85)	0.83	5.04 (±0.96)	5.03 (±1.05)	0.98
Suprailiac skinfolds, mm	3.70 (±0.95)	3.72 (±0.92)	0.98	4.35 (±0.94)	4.24 (±0.92)	0.37	4.22 (±0.95)	4.24 (±0.98)	0.87
Triceps skinfolds, mm	6.08 (±1.27)	6.22 (±1.27)	0.58	6.78 (±1.69)	6.45 (±1.32)	0.07	6.68 (±1.54)	6.66 (±1.36)	0.54
Biceps skinfolds, mm	4.59 (±1.09)	4.41 (±1.07)	0.38	4.99 (±1.19)	5.03 (±1.07)	0.34	4.59 (±1.07)	4.86 (±1.34)	0.07

^1^ Data are reported as means (±SD). Statistical significance level *p* < 0.05. ^2^ Comparison between means at baseline. ^3^ Tukey pairwise comparisons, treatment, and week interaction.

## Data Availability

The raw data supporting the conclusions of this article will be made available by the authors on request.

## Supplementary Materials

The following supporting information can be downloaded at: https://www.mdpi.com/article/10.3390/nu18121836/s1, Supplementary Table S1: RUTF ingredient and nutritional profile; Supplementary Table S2: Four skinfold body composition prediction equations adjusted for children’s height; Supplementary Table S3: Infant and Child Feeding Index (ICFI) scoring table; Supplementary Table S4: Average percent RUTF consumption weeks 1–8, 1–4, and 5–8 by treatment and age group; Supplementary Table S5. RUTF consumption dose according to children’s body weight for SEHAT RCT; Supplementary Table S6. Infant and Child Feeding Index (ICFI) analysis at weeks 8 and 16; Supplementary Table S7. FMI and FFMI analysis; Tukey’s comparison of the week-by-treatment group analysis; Supplementary Table S8. Baseline characteristics of dropouts by treatment arm; Supplementary Table S9. ITT-LOCF FMI and FFMI analysis; Tukey’s comparison of the week-by-treatment group analysis; Supplementary Table S10. Body composition in children aged 6 to 59 months at baseline, week 8, and week 16 of the study, and ITT-LOCF analysis; Supplementary Figure S1: Skinfold differences and each time point compared to baseline; Supplementary Figure S2: Body composition outcome, BFP, FM, FFM differences, and each time point compared to baseline; Supplementary Material S1: SEHAT study protocol: Solutions to Enhance Health with Alternative Treatment (SEHAT) protocol: a double-blinded randomized controlled trial for gut microbiota-targeted treatment of severe acute malnutrition using rice bran in ready-to-use therapeutic foods in Indonesia.

## Abbreviations

The following abbreviations are used in this manuscript:

BFP	Body Fat Percentage
FFM	Fat-Free Mass
FFMI	Fat-Free Mass Index
FM	Fat Mass
FMI	Fat Mass Index
GMP	Good Manufacturing Practices
HAZ	Height-For-Age Z-Score
IQR	Interquartile Range
ITT	Intention-To-Treat
IYCF	Infant and Young Child Feeding
LMIC	Low- and Middle-Income Countries
LOCF	Last Observation Carried Forward
MHREC	Medical And Health Research Ethical Committee
MUAC	Mid-Upper Arm Circumference
NCDs	Non-Communicable Diseases
PP	Per-Protocol
RUTF	Ready-To-Use Therapeutic Foods
SAM	Severe Acute Malnutrition
SE	Standard Error
SEAFAST	South East Asia Food And Agriculture Science And Technology
SEHAT	Solution To Enhance Health With Alternative Treatment
SD	Standard Deviation
WAZ	Weight-For-Age Z-Score
WHZ	Weight-For-Height Z-Score
WHO	World Health Organization
