# Supplementary material for: Addition of Prebiotic Rice Bran to Ready-to-Use Therapeutic Food Modulated Changes in Body Composition Only of 6–23-Month-Old Children During Treatment for Uncomplicated Acute Malnutrition: The Solutions to Enhance Health with Alternative Treatment (SEHAT) Study
Source: Nutrients. 2026 Jun 6;18(12):1836. doi: 10.3390/nu18121836 (PMC13305828; doi:10.3390/nu18121836)
Supplement: Supplementary file 1 [file nutrients-18-01836-s001.zip › Supplementary Figures.pdf]

Fig.1 Skinfolds differences, each time point compared to baseline

A.

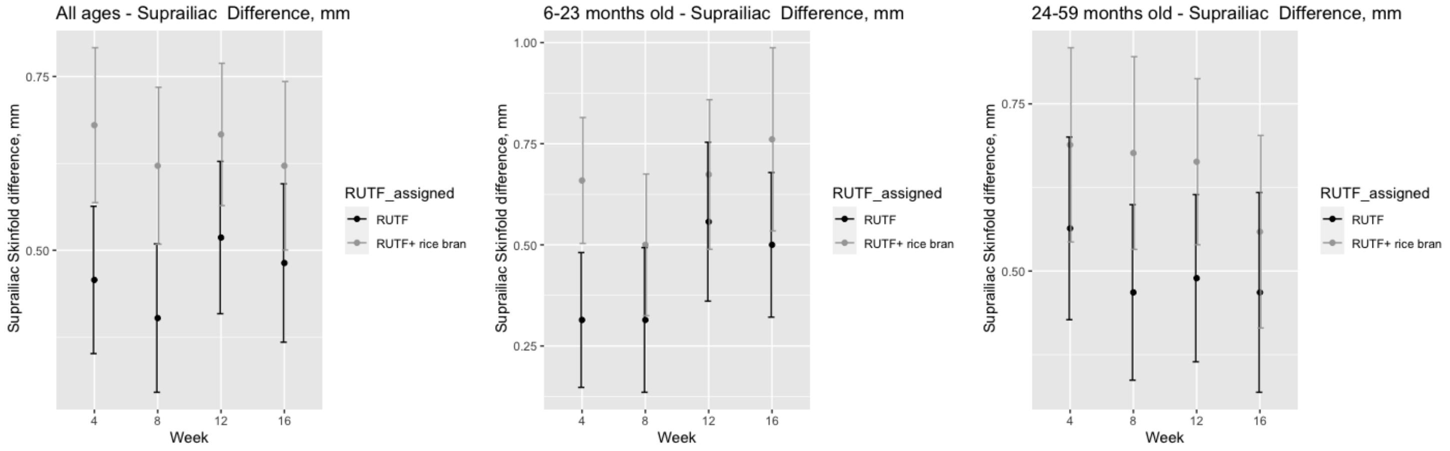

B.

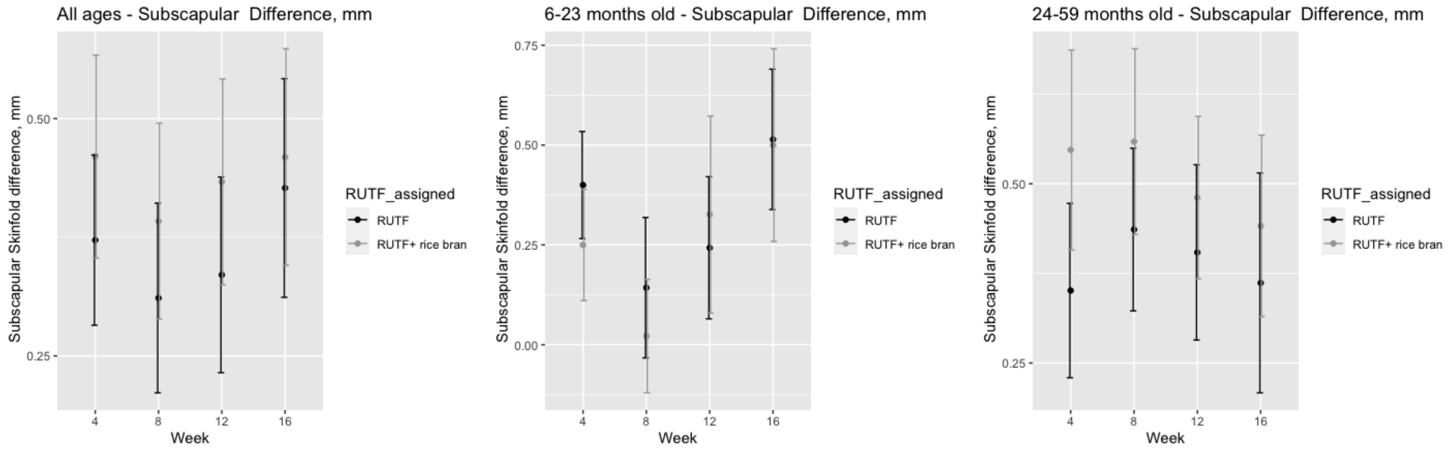

C.

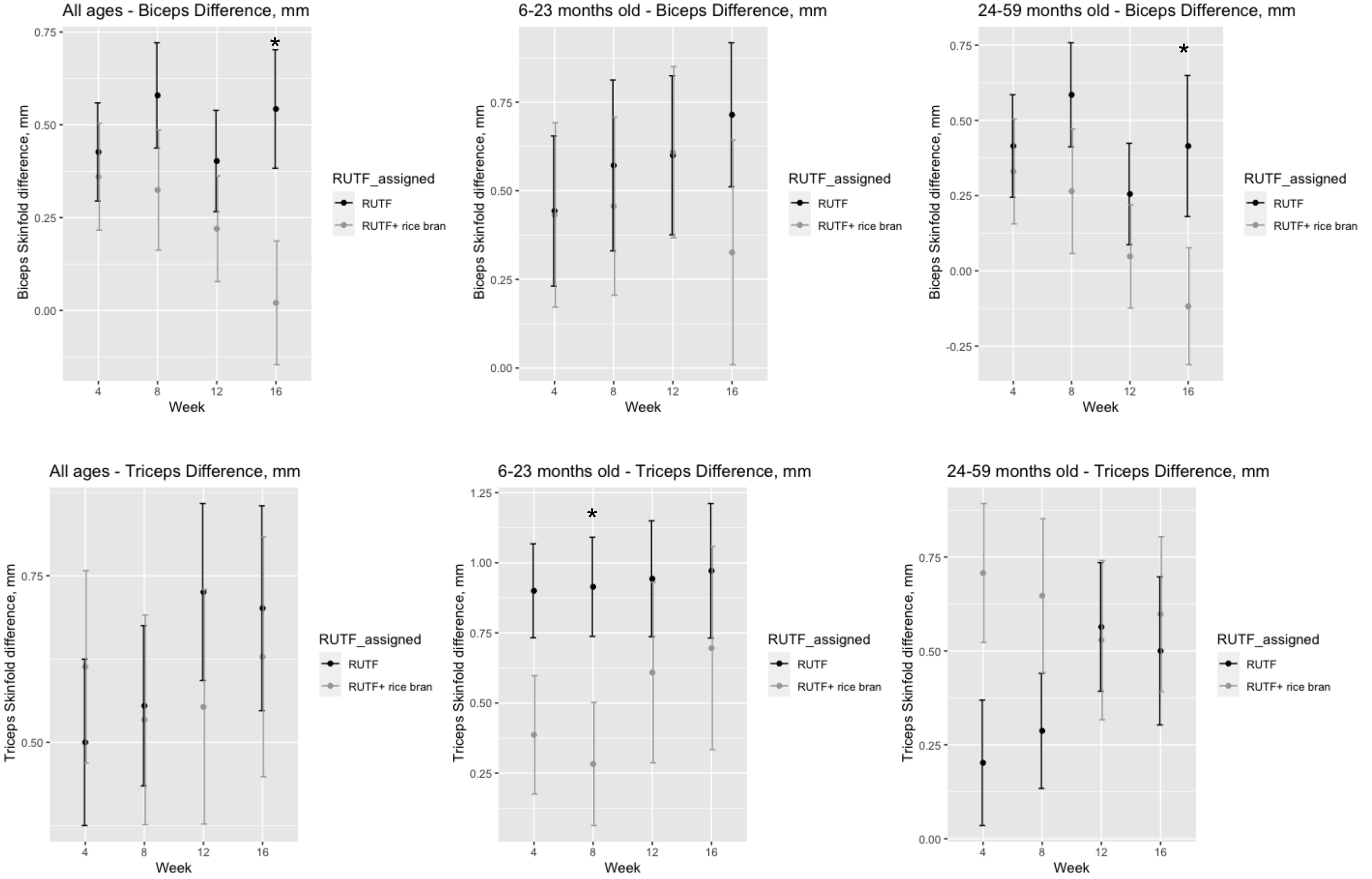

All ages - Biceps Difference, mm
6-23 months old - Biceps Difference, mm
24-59 months old - Biceps Difference, mm
Biceps Skinfold difference, mm
Week
RUTF_assigned
RUTF
RUTF+ rice bran
All ages - Triceps Difference, mm
6-23 months old - Triceps Difference, mm
24-59 months old - Triceps Difference, mm
Triceps Skinfold difference, mm

E.

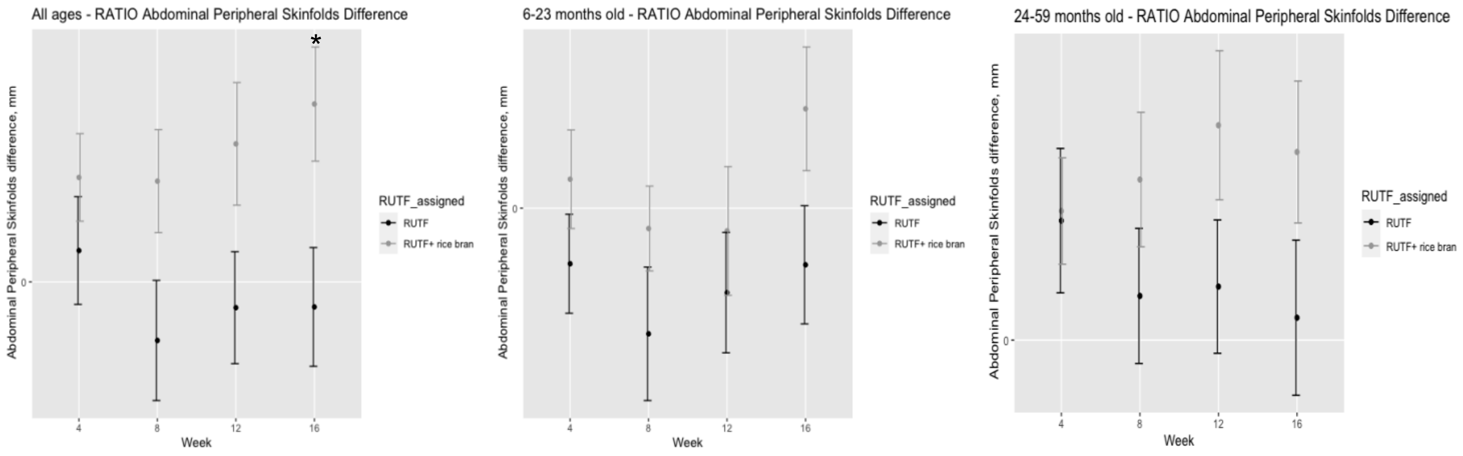

Fig. 2 Body composition outcome, BFP, FM, FFM differences, each time point compared to baseline.

A.

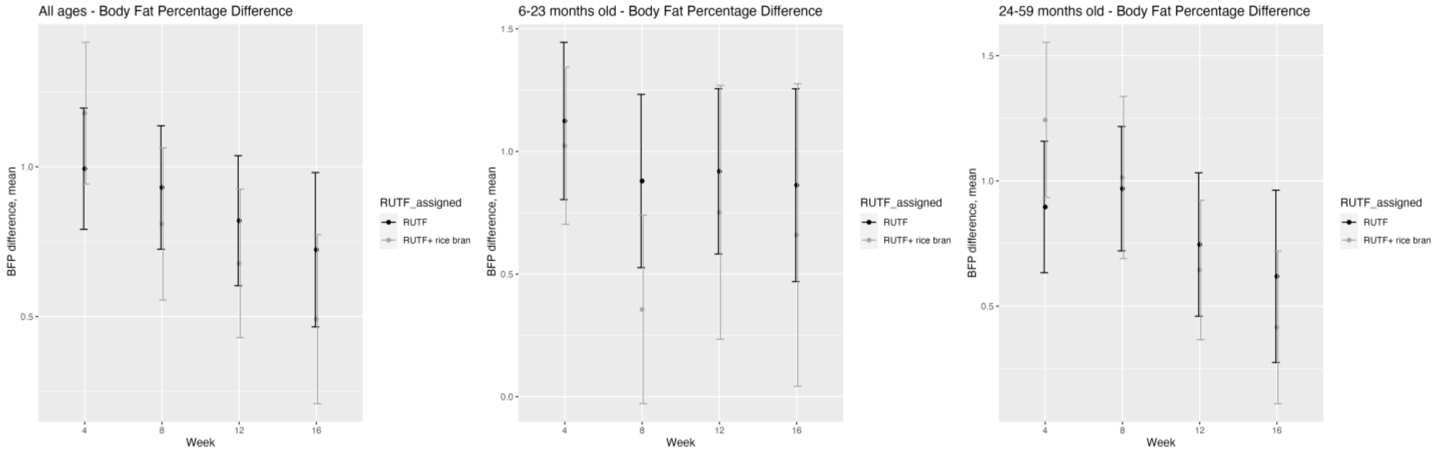

B.

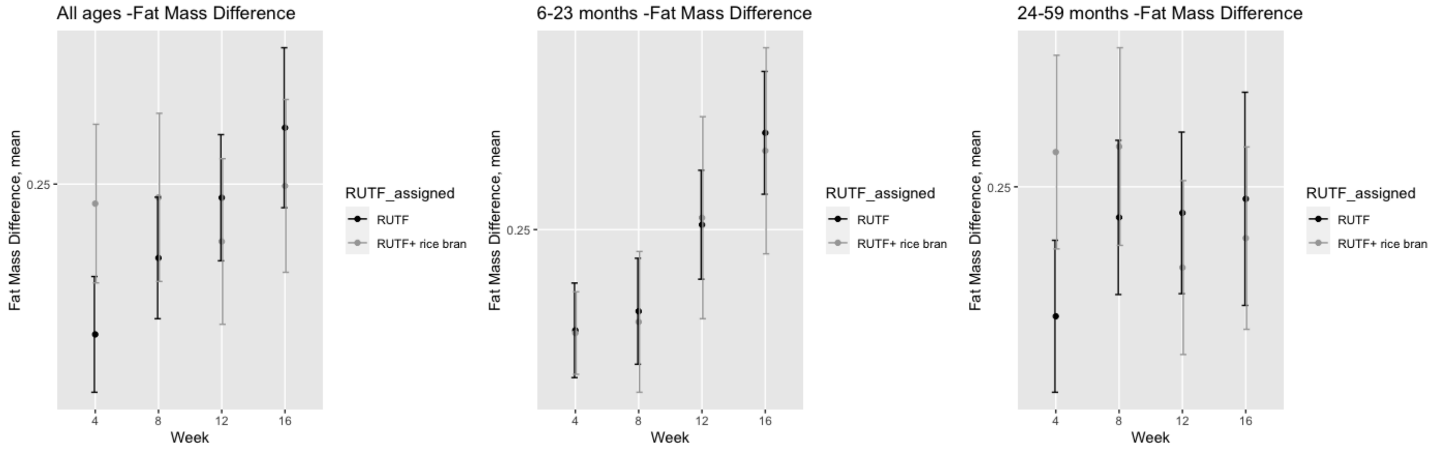
All ages -Fat Mass Difference
6-23 months -Fat Mass Difference
24-59 months -Fat Mass Difference
Fat Mass Difference, mean
0.25
Week
4
8
12
16
RUTF_assigned
RUTF
RUTF+ rice bran

C.

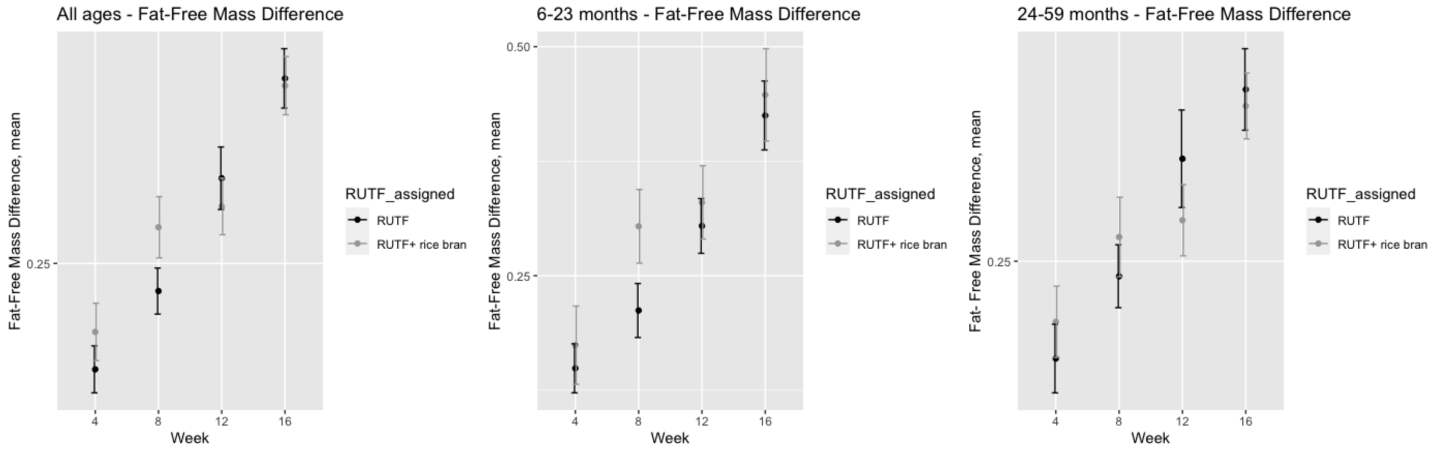
All ages - Fat-Free Mass Difference
6-23 months - Fat-Free Mass Difference
24-59 months - Fat-Free Mass Difference
Fat-Free Mass Difference, mean
Fat- Free Mass Difference, mean
0.25
0.50
Week
4
8
12
16
RUTF_assigned
RUTF
RUTF+ rice bran
