# Supplementary material for: Addition of Prebiotic Rice Bran to Ready-to-Use Therapeutic Food Modulated Changes in Body Composition Only of 6–23-Month-Old Children During Treatment for Uncomplicated Acute Malnutrition: The Solutions to Enhance Health with Alternative Treatment (SEHAT) Study
Source: Nutrients. 2026 Jun 6;18(12):1836. doi: 10.3390/nu18121836 (PMC13305828; doi:10.3390/nu18121836)
Supplement: Supplementary file 1 [file nutrients-18-01836-s001.zip › Supplementary Tables.pdf]

Supplemental Tables

Supplemental Table S1. RUTF Ingredient and Nutritional Profile

	RUTF-Rice Bran	RUTF
**Ingredients, g/100 g**		
Palm oil	20.8	21.7
Whole milk powder	18.1	17.6
Peanut butter	8.6	8.6
Sugar	14.4	15.0
Whey protein concentrate	8.0	7.9
Skim milk powder	4.0	6.7
Wheat flour	8.7	9.2
Rice flour	8.4	8.7
Maltodextrin	0.1	0.7
Vitamin and minerals premix	2.0	2.0
Rice bran	5.0	-
Cocoa powder/vanilla	1.4	1.4
Proprietary ingredients (binder)	0.5	0.5
**Nutrient composition, g/100 g**		
Energy, kcal/100g	520	534
Protein	15.0	13.5
Fat	31.1	31.6
Carbohydrate	44.4	49.0
Fibre	2.0	1.5
Protein-Energy Ratio	11.5	10.1
Fat-Energy Ratio	53.8	53.2

Supplemental Table S2. **Four Skinfolds Body composition prediction equations adjusted for children's height**

Sex	Body Composition Equations
Females	(13·99*log Sum 4SF) − (21·42*Log Height) +85·65
Males	(12·74*log Sum 4SF) − (21.47*Log Height) +87·82

Supplemental Table S3. **Infant and Child Feeding Index (ICFI) scoring table**

	**Age Group (Months)**							
	6-8		9-11		12-35		36-59	
	**Value**	**Score**	**Value**	**Score**	**Value**	**Score**	**Value**	**Score**
**Breastfed** (24 hours)	Yes	+2	Yes	+2	Yes	+1	Yes	0
**Food Groups** (24 hours)	1<br>≥2	+1<br>+2	1 or 2<br>≥3	+1<br>+2	2 or 3<br>≥4	+1<br>+2	3 or 4<br>≥5	+2<br>+3
**Meal frequency** (24 hours)	1<br>≥2	+1<br>+2	1 or 2<br>≥3	+1<br>+2	2<br>3<br>≥4	+1<br>+2<br>+3	2<br>3<br>≥4	+1<br>+2<br>+3

Supplemental Table S4. **Average percent RUTF consumption week 1-8, 1-4, and 5-8 by treatment and age group.**

	**All-ages**					**6-23m**					**24-59m**				
**Treatment**	**N**	**Mean**	**Sd**	**SE**	**p-value**	**N**	**Mean**	**Sd**	**SE**	**p-value**	**N**	**Mean**	**Sd**	**SE**	**p-value**
**Week 1-8**															
RUTF+ rice bran	74	21·23	17·26	2·01	0·91	26	24·63	16·38	3·21	0·23	48	19·38	17·62	2·54	0·45
RUTF	82	20·94	14·22	1·57		35	19·72	14·55	2·46		47	21·85	14·06	2·05	
**Week 1-4**															
RUTF+ rice bran	75	23·60	18·97	2·19	0·85	25	29·11	20·37	4·07	0·19	50	20·84	17·80	2·52	0·22
RUTF	82	24·15	17·16	1·89		35	22·59	16·52	2·79		47	25·31	17·71	2·58	
**Week 5-8**															
RUTF+ rice bran	74	18·31	17·16	1·99	0·81	26	20·04	14·90	2·92	0·40	48	17·38	18·35	2·65	0·76
RUTF	82	17·73	13·08	1·44		35	16·86	13·78	2·33		47	18·38	12·64	1·84	

Supplemental Table S5. RUTF consumption dose according to children's body weight for SEHAT RCT

Child's Weight (Kg)	Sachet per day	Sachet per week	Kcal per day
4.0-4.9	1 ½	10	750
5.0-6.9	2	15	1,000
7.0-9.9	3	20	1,500
10.0-14.9	4	30	2,000

***Supplemental Table S6*. Infant and Child Feeding Index (ICFI) analysis at weeks 8 and 16**

	Week 8			Week 16		
	RUTF+ rice bran	RUTF	P value	RUTF+ rice bran	RUTF	P value
**ICFI**			0·60[1]			0·70[1]
Acceptable, n(%)	51 (69)	56 (70)		59 (80)	62 (77)	
Borderline, n(%)	19 (26)	17 (21)		11 (15)	16 (20)	
Poor, n(%)	4 (5·4)	7 (8·8)		4 (5·4)	3 (3·7)	
Unknown, n(%)	0	2		0	1	
**Breastfeeding,** n(%)	14 (19)	24 (29)	0·13[2]	6 (55)	15 (52)	0·90[2]

***Supplemental Table S7*. FMI and FFMI analysis, Tukey comparison of the week-by-treatment group analysis**

**FMI**	**All ages**		**6-23 months**		**24-59 months**	
Contrast	RUTF+ rice bran	RUTF	RUTF+ rice bran	RUTF	RUTF+ rice bran	RUTF
week0 - week4	<·0001	<·0001	0·04	0·001	<·0001	0·0001
week0 - week8	<·0001	<·0001	0·48	0·02	<·0001	<·0001
week0 - week12	<·0001	<·0001	0·27	0·04	0·0006	<·0001
week0 - week16	<·0001	<·0001	0·29	0·04	0·0013	0·0002
week4 - week8	0·72	1	0·77	0·90	0·92	0·94
week4 - week12	0·05	1	0·94	0·84	0·01	1
week4 - week16	0·07	1	1	1	0·01	1
week8 - week12	0·59	1	1	1	0·13	0·96
week8 - week16	0·65	1	1	1	0·09	0·89
week12 - week16	1	1	1	1	0·99	0·99
**FFMI**	**All ages**		**6-23 months**		**24-59 months**	
Contrast	RUTF+ rice bran	RUTF	RUTF+ rice bran	RUTF	RUTF+ rice bran	RUTF
week0 - week4	0·01	0·45	0·83	0·97	0·006	0·39
week0 - week8	0·01	0·64	0·18	1	0·11	0·29
week0 - week12	0·99	0·99	0·97	0·97	1	0·59
week0 - week16	0·89	0·99	0·86	1	0·97	0·92
week4 - week8	0·99	0·99	0·78	0·99	0·88	1
week4 - week12	0·02	0·69	1	0·74	0·007	1
week4 - week16	0·15	0·56	1	0·95	0·06	0·89
week8 - week12	0·03	0·84	0·52	0·93	0·11	0·99
week8 - week16	0·19	0·74	0·81	1	0·40	0·81
week12 - week16	0·95	0·99	0·99	1	0·97	0·97

Results are averaged over sex. P value adjustment: tukey method for comparing a family of 5 estimates.

Suplemental Table S8 . Baseline characteristics of dropouts by treatment arm

**Characteristic**	**Overall**, N = 44[1]	**RUTF+ rice bran**, N = 21[1]	**RUTF**, N = 23[1]	**p-value**[2]
**Age**	31.01 (14.85)	33.10 (17.09)	29.10 (12.55)	0.6
**Sex**				0.3
Female	20 (45%)	8 (38%)	12 (52%)	
Male	24 (55%)	13 (62%)	11 (48%)	
**Age group**				0.6
24-59m	27 (61%)	12 (57%)	15 (65%)	
6-23m	17 (39%)	9 (43%)	8 (35%)	
**WHZ**	-3.00 (0.43)	-2.99 (0.51)	-3.01 (0.34)	0.5
**HAZ**	-2.66 (0.86)	-2.52 (0.80)	-2.79 (0.91)	0.3
**WAZ**	-3.50 (0.50)	-3.39 (0.49)	-3.60 (0.50)	0.11

Characteristic	**Overall**, N = 44[1]	**RUTF+ rice bran**, N = 21[1]	**RUTF**, N = 23[1]	**p-value**[2]
**MUAC**	12.78 (0.64)	12.80 (0.65)	12.75 (0.65)	0.8
**MUACZ**	-2.29 (0.50)	-2.35 (0.58)	-2.24 (0.42)	>0.9
**FM**	2.63 (0.39)	2.71 (0.44)	2.55 (0.33)	0.3
**FFM**	5.88 (1.44)	6.14 (1.57)	5.64 (1.30)	0.3
**BFP**	31.36 (3.24)	31.12 (3.49)	31.57 (3.05)	0.5
**Acute Malnutrition**				0.5
MAM	27 (61%)	14 (67%)	13 (57%)	
SAM	17 (39%)	7 (33%)	10 (43%)	
**Stunting**	13 (30%)	5 (24%)	8 (35%)	0.4
**Edema**	1 (2.3%)	0 (0%)	1 (4.3%)	>0.9

[1] Mean (SD); n (%)

[2] Wilcoxon rank sum test; Pearson's Chi-squared test; Fisher's exact test

Supplementary Table S9. ITT-LOCF FMI and FFMI analysis, Tukey comparison of the week-by-treatment group analysis

**FMI**	**All ages**		**6-23 months**		**24-59 months**	
Contrast	RUTF+ rice bran	RUTF	RUTF+ rice bran	RUTF	RUTF+ rice bran	RUTF
week0 - week4	<.0001	<.0001	0.7802	0.9943	0.0053	0.3659
week0 - week8	<.0001	<.0001	0.2583	0.9995	0.0784	0.2517
week0 - week12	0.0019	0.0001	0.9960	0.7308	0.9999	0.4934
week0 - week16	0.0088	0.0010	0.9636	0.8838	0.9196	0.8271
week4 - week8	0.5583	0.9926	0.9067	0.9709	0.8986	0.9995
week4 - week12	0.0030	0.6194	0.9367	0.4733	0.0085	0.9996
week4 - week16	0.0006	0.3134	0.9888	0.6679	0.0673	0.9430
week8 - week12	0.2215	0.8652	0.4660	0.8488	0.1105	0.9931
week8 - week16	0.0863	0.5799	0.6562	0.9537	0.4162	0.8671
week12 - week16	0.9932	0.9878	0.9983	0.9982	0.9577	0.9822
**FFMI**	**All ages**		**6-23 months**		**24-59 months**	
Contrast	RUTF+ rice bran	RUTF	RUTF+ rice bran	RUTF	RUTF+ rice bran	RUTF
week0 - week4	0.0063	0.6673	0.7802	0.9943	0.0053	0.3659
week0 - week8	0.0105	0.9975	0.2583	0.9995	0.0784	0.2517
week0 - week12	0.9971	0.9999	0.9960	0.7308	0.9999	0.4934
week0 - week16	0.8254	0.9988	0.9636	0.8838	0.9196	0.8271
week4 - week8	0.9999	0.7020	0.9067	0.9709	0.8986	0.9995
week4 - week12	0.0191	0.5785	0.9367	0.4733	0.0085	0.9996
week4 - week16	0.1331	0.8503	0.9888	0.6679	0.0673	0.9430
week8 - week12	0.0302	0.7488	0.4660	0.8488	0.1105	0.9931
week8 - week16	0.1850	0.9997	0.6562	0.9537	0.4162	0.8671
week12 - week16	0.9512	0.6673	0.9983	0.9982	0.9577	0.9822

Results are averaged over sex. Degrees-of-freedom method: kenward-roger

P value adjustment: tukey method for comparing a family of 5 estimates.

Supplementary Table S10. A. Body composition in children aged 6 to 59 months at baseline, week 8 and week 16 of the study, ITT-LOCF analysis

	Week 8			Week 16		
Outcome	RUTF+ rice bran	RUTF	***p-value***[3]	RUTF+ rice bran	RUTF	***p-value***[3]
BFP, %	31.7 (2.98)	31.8 (3.07)	0.23	31.5 (3.07)	31.7 (3.29)	0.40
FM, Kg	2.87 (0.49)	2.77 (0.49)	0.53	2.88 (0.45)	2.81 (0.46)	0.75
FFM, Kg	6.27 (1.40)	6.07 (1.60)	0.21	6.37 (1.40)	6.21 (1.61)	0.08
FMI, Kg/m^2	4.07 (0.50)	4.08 (0.49)	0.22	4.00 (0.52)	4.04 (0.53)	0.54
FFMI, Kg/m^2	8.74 (0.51)	8.73 (0.49)	0.88	8.68 (0.50)	8.69 (0.48)	0.51
Ratio Abdominal vs Peripheral Skinfolds	0.82 (0.15)	0.81 (0.16)	0.51	0.84 (0.17)	0.81 (0.16)	0.25
Sub-scapular Skinfolds, mm	4.95 (0.98)	4.74 (0.97)	0.19	5.01 (0.90)	4.83 (1.02)	0.27
Suprailiac Skinfolds, mm	4.16 (1.02)	3.99 (0.94)	0.27	4.13 (0.95)	4.05 (0.94)	0.68
Triceps Skinfolds, mm	6.49 (1.63)	6.26 (1.39)	0.44	6.55 (1.61)	6.38 (1.48)	0.63
Biceps Skinfolds, mm	4.83 (1.09)	4.80 (1.11)	0.51	4.61 (1.04)	4.78 (1.23)	0.25

Supplementary Table S10.B. Body composition in children aged 6 to 23 months at baseline, week 8 and week 16 of the study, ITT-LOCF analysis

	**Week 8**			**Week 16**		
Outcome	RUTF+ rice bran	RUTF	***p-value***[3]	RUTF+ rice bran	RUTF	***p-value***[3]
BFP, %	33.6 (2.07)	33.7 (2.36)	0.90	33.8 (2.38)	33.6 (2.37)	0.72
FM, Kg	2.46 (0.35)	2.40 (0.31)	0.88	2.53 (0.35)	2.49 (0.32)	0.71
FFM, Kg	4.88 (0.71)	4.75 (0.62)	0.98	4.99 (0.78)	4.93 (0.66)	0.45
FMI, Kg/m^2	4.42 (0.36)	4.44 (0.36)	0.78	4.44 (0.37)	4.42 (0.36)	0.90
FFMI, Kg/m^2	8.76 (0.55)	8.75 (0.47)	0.78	8.69 (0.53)	8.72 (0.48)	0.90
Ratio Abdominal vs Peripheral Skinfolds	0.78 (0.16)	0.80 (0.15)	0.68	0.82 (0.20)	0.83 (0.14)	0.96
Sub-scapular Skinfolds, mm	4.52 (0.89)	4.55 (0.92)	0.57	4.87 (0.89)	4.86 (0.91)	0.72
Suprailiac Skinfolds, mm	3.89 (1.11)	3.87 (0.83)	0.95	4.08 (1.00)	4.02 (0.76)	0.91
Triceps Skinfolds, mm	6.16 (1.53)	6.00 (1.49)	0.85	6.47 (1.73)	6.05 (1.64)	0.54
Biceps Skinfolds, mm	4.84 (0.91)	4.74 (1.09)	0.93	4.74 (1.02)	4.86 (1.09)	0.44

Supplementary Table S10.C. Body composition in children aged 24 to 59 months at baseline, week 8 and week 16 of the study, ITT-LOCF analysis

	Week 8			Week 16		
Outcome	RUTF+ rice bran	RUTF	***p-value***[3]	RUTF+ rice bran	RUTF	***p-value***[3]
BFP, %	30.8 (2.96)	30.6 (2.89)	0.18	30.3 (2.72)	30.0 (3.16)	0.49
FM, Kg	3.08 (0.43)	3.02 (0.43)	0.48	3.04 (0.39)	3.02 (0.41)	0.91
FFM, Kg	6.95 (1.12)	6.95 (1.43)	0.21	7.04 (1.12)	7.06 (1.48)	0.15
FMI, Kg/m^2	3.90 (0.47)	3.84 (0.41)	0.79	3.78 (0.45)	3.78 (0.46)	0.55
FFMI, Kg/m^2	8.73 (0.50)	8.71 (0.50)	0.79	8.67 (0.49)	8.68 (0.49)	0.55
Ratio Abdominal vs Peripheral Skinfolds	0.84 (0.14)	0.81 (0.17)	0.24	0.85 (0.16)	0.80 (0.17)	0.12
Sub-scapular Skinfolds, mm	5.16 (0.96)	4.87 (0.99)	0.09	5.08 (0.91)	4.82 (1.10)	0.12
Suprailiac Skinfolds, mm	4.29 (0.96)	4.07 (1.00)	0.16	4.15 (0.94)	4.07 (1.05)	0.58
Triceps Skinfolds, mm	6.66 (1.66)	6. 44 (1.31)	0.40	6.59 (1.56)	6.60 (1.34)	0.97
Biceps Skinfolds, mm	4.83 (1.18)	4.85 (1.13)	0.91	4.54 (1.06)	4.72 (1.31)	0.36
